# Synthesis and plugging behavior of fluorescent polymer microspheres as a kind of conformance control agent in reservoirs

**DOI:** 10.1039/c8ra00903a

**Published:** 2018-03-15

**Authors:** Hongbin Yang, Leilei Hu, Chao Chen, Yongbo Gao, Xuechen Tang, Xia Yin, Wanli Kang

**Affiliations:** School of Petroleum Engineering, China University of Petroleum (East China) Qingdao 266580 People's Republic of China kangwanli@126.com; School of Environmental and Municipal Engineering, North China University of Water Resources and Electric Power Zhengzhou 450045 People's Republic of China huleilei449@163.com

## Abstract

The fluorescent polymer microsphere is a newly developed chemical agent for conformance control in reservoirs. In this paper, one kind of fluorescent polymer microspheres P(AM-BA-RhB) was synthesized *via* the inverse suspension polymerization method with Rhodamine B as a fluorescence functional monomer. Laboratory experiments were performed to characterize the morphology, fluorescent property, swelling property and plugging behavior of fluorescent polymer microspheres. The experimental results showed that the polymer microspheres P(AM-BA-RhB) displayed stable fluorescence performance in solutions containing metal ions at pH values between 3.0 and 10.0. The swelling property was not dramatically affected by the Rhodamine B embedded in the polymer microspheres by grafting. Both a visual micromodel test and sand-pack tubes experiment demonstrated that the fluorescent polymer microspheres could pass directly or by deformation through porous media and get into the in-depth formation. The injection pressure showed the phenomenon of “Wave-type Variation”. Three plugging behaviors such as piston plugging, protruding plugging and fingering plugging were put forward. The introduction of fluorescent polymer microspheres could provide one method to research the conformance control and EOR mechanism of polymer microspheres in the reservoirs.

## Abstract 2

Three plugging behavior such as piston plugging, protruding plugging and fingering plugging were put forward.

## Graphical abstract

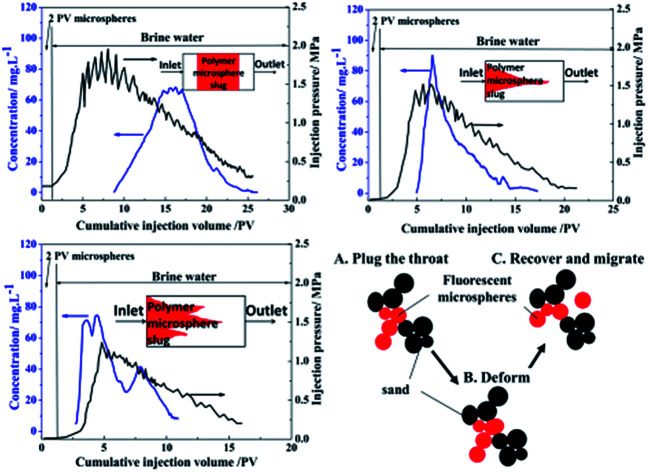

## 1. Introduction

According to previous reports, reservoirs with induced fractures or high permeability channels, commonly called thief zones or streaks due to extensive water flooding, are quite common in mature oil reservoirs. Low efficiency of the injected water, which would lead to excessive water production and rapid production decline, has become one of the most crucial problems in the late stage of development for mature oilfields.^1–3^ Therefore, it is important for the petroleum industry to develop more reliable techniques such as “green” water shut-off or conformance control. Gels have been introduced as water plugging agents to mitigate this problem.^1,4–8^ The gel application plug fractures and redirects water from high permeable zones to low-permeability areas. A new trend in gel treatments is using polymer microspheres since they can overcome some distinct drawbacks inherent in *in situ* gelation systems.^9–12^ The polymer microsphere is a kind of viscoelastic conformance control agent with three-dimensional structure and can absorb the formation water and migration in the porous medium of the reservoirs under the injection pressure.^13–15^ Field test results in Shengli, Dagang and Jidong Oilfields in China showed that polymer microsphere was a promising conformance control agent in the development of heterogeneous reservoirs, especially the fractured reservoirs.^16–18^ Hua and Lin *et al.* investigated the shape, size, rheological properties, plugging properties, profile control mechanism and oil displacement mechanism of the nanoscale polymer microspheres. They found that polymer microspheres could reduce water permeability because the microspheres adsorbed, accumulated and bridged in the pore throat, and the adsorbed layers would be collapsed under the pressure, entering deep into the reservoir due to the good deformation properties of the microspheres.^13^ Yao and Wang *et al.* researched the effects of ionic strength on the transport and retention of polyacrylamide microspheres in porous media.^19^ Yang and Kang *et al.* researched the mechanism and influencing factors on the initial particle size and swelling capability of polymer microspheres from the synthesis and reservoir condition.^20^ Yang and Xie *et al.* optimized the injection parameters of polymer microspheres and polymer composite flooding system, and they found that the composite system could better improve polymer flooding at the displacement rate of 3.5 m d^−1^ and the injection volume of 530 mg L^−1^ PV.^21^

During the application of polymer microspheres, polymer solution was injected into the reservoir along with polymer microspheres.^21,22^ Due to the same amide functional groups in polymer microspheres and polymer solutions, the conventional concentration method of polymer microspheres such as starch–cadmium iodide method, chemiluminescent nitrogen method, ammonia electrode method and organic carbon content method were not accurate.^23–25^

So it is difficult to research the plugging behavior and conformance control mechanism from a quantitative perspective. For these reasons, fluorescent polymer microspheres could overcome the limits of conventional methods and achieve the real-time detection of polymer microspheres concentration and the distribution of polymer microspheres in the reservoir during the flooding process. Fluorescent polymer microspheres have a dual function, one is the conformance agent and the other is the oil field tracer. The oil field tracer will be a new application of fluorescent polymer microspheres in the development of oilfields.

Under most circumstances, fluorescent polymer microspheres are physically dyed through absorption or embedment, which results to the problem of dye leakage and limits the field applications of these fluorescent polymer microspheres. To avoid dye leakage, dyes could be covalently incorporated into polymer microsphere by the inverse suspension polymerization.^26–28^ Rhodamine B (RhB), which has been widely used in medicine, environmental protection, textiles, colored glass, and cosmetics *etc.*, is a commonly used commercial fluorescent dye.^29–32^

Herein, we report one kind of fluorescent polymer microspheres P(AM-BA-RhB) acted as a novel conformance control agent. The fluorescent polymer microspheres were covalently dyed with Allyl Rhodamine B by inverse suspension polymerization. The polymer microsphere was characterized by fluorescence microscope, environmental scanning electron microscope (ESEM) and scanning electronic microscopy (SEM). The effects of salinity and temperature on the swelling property of the polymer microsphere were studied by weighing method. In addition, in order to define the scope of application, the fluorescence spectroscopy was used to investigate the effects of pH and ionic species on the fluorescence intensity of the polymer microsphere. In order to monitor the concentration of fluorescent polymer microspheres, the relationship between fluorescence intensity and concentration of fluorescent polymer microspheres was established. On the basis of these results, the plugging behavior of the polymer microsphere was investigated using core flooding test and visual micromodel. These researches provide theoretical support for the further study of fluorescent polymer microspheres in the application of oilfields.

## 2. Experimental section

### 2.1 Materials

Allyl Rhodamine B (RhB) was synthesized by the method illustrated in ref. 33. Sorbitan Monostearate (Span 60), purchased from Shanghai Shanpu Chemical Co., Ltd. (Shanghai, China), was used as an oil phase dispersant. Anhydrous sodium carbonate (Na_2_CO_3_), supplied by Shanghai Hongguang Chemical Plant (Shanghai, China), was employed as an electrolyte. *N*, *N*′-Methylenebisacrylamide (MBA) was purchased from Tianjin Kemiou Chemicals Ltd. (Tianjin, China) and used as a cross-linking agent. Ammonium persulfate (APS, used as water-soluble initiator), acrylamide (AM) monomer, anhydrous ethanol and other reagents used were all analytical-reagent grade, obtained from Sinopharm Chemicals Ltd. (Shanghai, China).

### 2.2 Synthesis and purification of fluorescent polymer microspheres

**Fig. 1:**
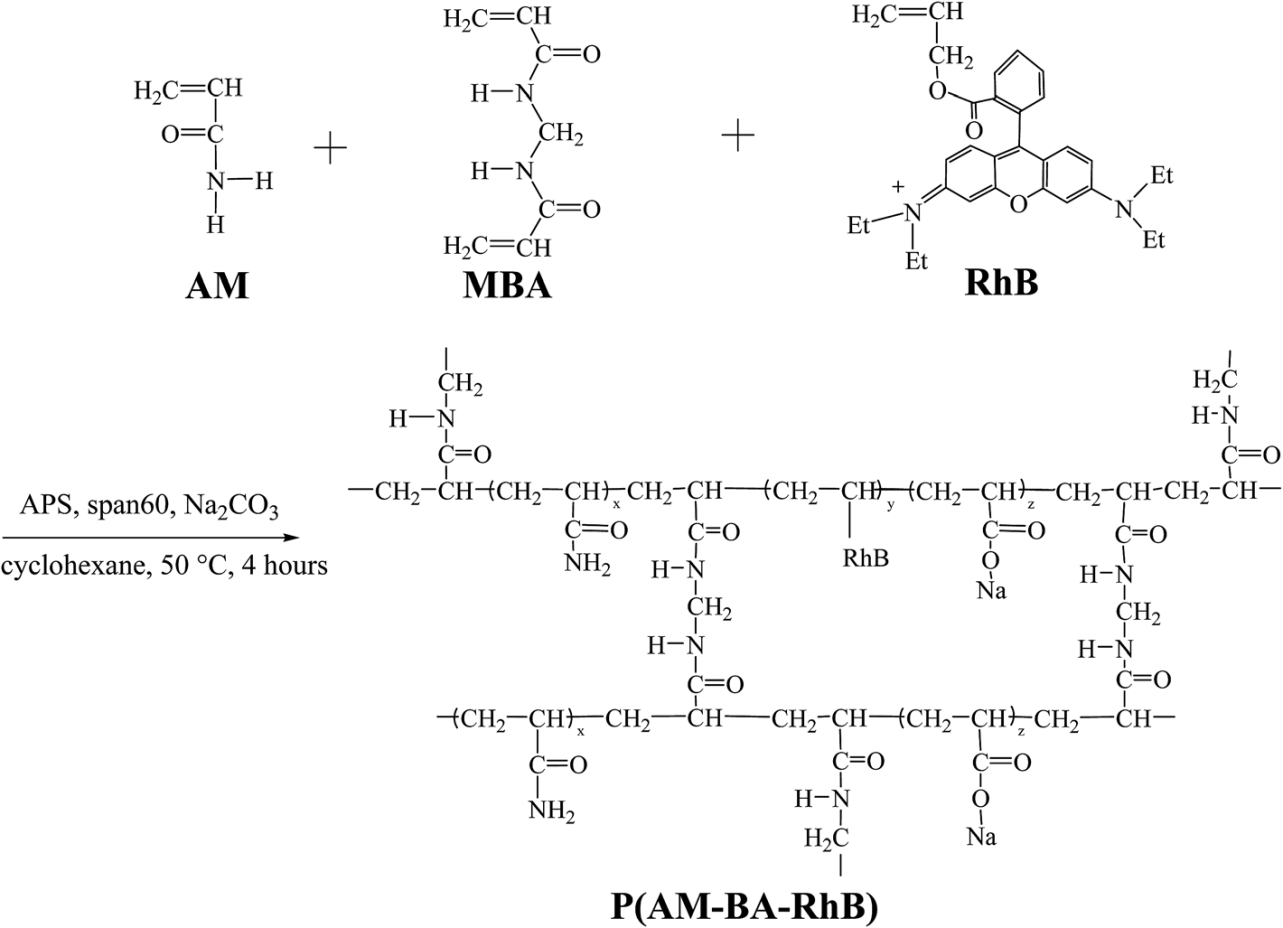
Synthesis route of fluorescent polymer microspheres P(AM-BA-RhB).

The polymerization was conducted in a 250 mL four-neck flask. The flask was equipped with mechanical stirrer, reflux condenser and a constant temperature water bath. The fluorescent polymer microsphere P(AM-BA-RhB) was prepared *via* inverse suspension polymerization using cyclohexane as the continuous phase and Span-60 as the nonionic polymeric surfactant (Fig. 1). 0.07 g MBA, 14.06 g AM, and Na_2_CO_3_ were dissolved in 40 mL distilled water; this solution was immediately poured into 60 mL of cyclohexane (containing 0.005 g Allyl Rhodamine B and 0.45 g Span 60) which was previously purged with dry nitrogen steam in the flask. Air was flushed from the reactor by introducing nitrogen until the entire process completed. After aqueous droplets in continuous phase appeared, 10 mL APS aqueous solution (75 g L^−1^) was added to continuous phase to initiate polymerization. The polymerization lasted for 4 h at 50 °C. After the prescribed time, fluorescent polymer microsphere appeared when stirring was stopped. Then, the precipitates were washed with large amounts of anhydrous ethanol and filtered with qualitative filter paper. The product was dried in a vacuum oven at 45 °C for 24 h. The conventional polymer microsphere of P(AM-BA) was prepared by the same process as described above, without adding the Allyl Rhodamine B in the reaction system.

### 2.3 Characterization methods

FT-IR spectra of fluorescent polymer microsphere P(AM-BA-RhB), conventional polymer microsphere P(AM-BA), and acrylamide monomer were measured with a Nicolet model NEXUS670 spectrometer with samples prepared on KBr pellets.

The surface morphology of these fluorescent polymer microspheres was observed by inverse fluorescence microscope (Leica DMI 3000B, Germany) and scanning electron microscopy (SU8010, HITACHT, Japan).

The average particle size of fluorescent polymer microsphere before and after swelling was determined at room temperature by RISE-2006 laser particle size analyzer provided by Jinan Runzhi Technology Co., Ltd of China.

The fluorescence spectra of fluorescent polymer microsphere were obtained from the Fluoromax-4 spectrometer (Horiba JobinYvon, American).

Environment Scanning Electron Microscope (ESEM) images were taken by FEI Quanta 200 FEG (FEI Company, Holland) to observe the structure of fluorescent polymer microsphere after swelling.

To measure the swelling ratio of fluorescent polymer microspheres, a tea bag (*i.e.*, a 100 mesh nylon screen) containing pre-weighed dry samples was immersed entirely in brine water (1 g L^−1^ NaCl). After being hydrated in brine water for various times, the swollen fluorescent polymer microspheres were allowed to drain by taking the teabag from the brine, followed by removal of excess surface water using filter paper. The swelling ratio, *S*_w_ was calculated from the following equation:^20^

1 *S*_w_ = (*m*_1_ − *m*_0_)/*m*_0_

where *m*_0_ and *m*_1_ are the weights of the dry and swollen fluorescent polymer microspheres, respectively.

### 2.4 Plugging behavior experiments of fluorescent polymer microspheres

#### 2.4.1 Visual micromodel test

**Fig. 2:**
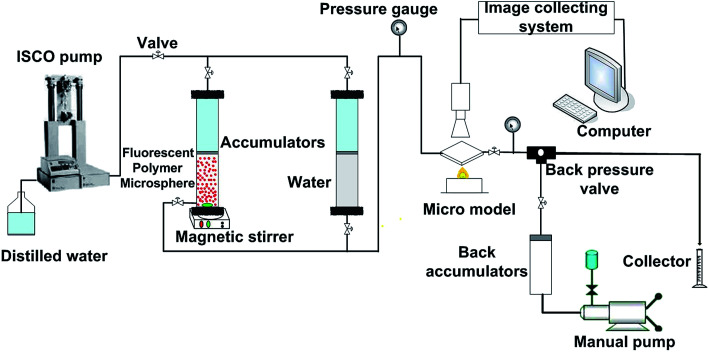
Experimental setup of the visual micromodel test.

The visual micromodel was used to study the migration mechanism of fluorescent polymer microspheres. The visual micromodel is a kind of etched glass model. The etched glass model was prepared by laser etching, and pore throat diameter from 300 μm to 400 μm. The whole size of the visual micromodel in the experiment was 6.3 cm × 6.3 cm × 0.4 cm. The actual internal porous model size was 4.0 cm × 4.0 cm × 0.4 cm. Two pores are located on the diagonal of the model to simulate the injection well and production well. The basic experimental setup is illustrated in Fig. 2. An accumulator with magnetic stirrer was used to contain the dispersed fluorescent polymer microspheres solution. The procedures of the experiment are as follows: (1) saturating the visual micromodel with brine water; (2) injecting the dispersed fluorescent polymer microspheres solution with concentration of 2000 mg L^−1^ into the visual micromodel, and the injection rate was 0.003 mL min^−1^. Record the dynamic process of polymer microspheres in the porous medium; (3) clean the visual micromodel; (4) analysis the images and conclude the migration law.

#### 2.4.2 Core flooding test

**Fig. 3:**
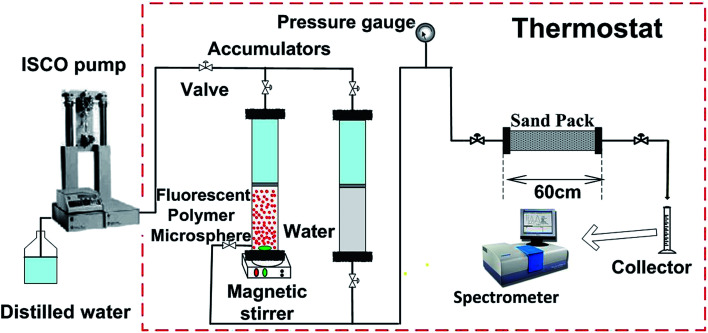
Flow diagram of the core flooding test.

The multifunction displacement device was used to study the plugging mechanism of fluorescent polymer microspheres. The experimental setup in this study (see Fig. 3) was constructed from a sand-pack 60 cm in length and 2.5 cm in diameter. It was packed with different sand grains to simulate the reservoir. An ISCO pump was used to inject the dispersed fluorescent polymer microspheres solution and brine water from accumulators to the sand-pack model. An accumulator with magnetic stirrer was used to contain the dispersed fluorescent polymer microspheres solution. Pressure gauge was mounted on the inlet to monitor the injection pressure during the whole injection progress. Cylinder mounted on the model outlet was used as a collector to record the volume of fluorescent polymer microspheres and brine water production. The Fluoromax-4 spectrometer was used to evaluate the fluorescence intensity and get the concentrations of fluorescent polymer microspheres at different stages. The injection concentration of fluorescent polymer microspheres P(AM-BA-RhB) used in this experiment was 2000 mg L^−1^ and experimental temperature was 50 °C. The procedures of the experiment are as follows: (1) vacuuming and saturating the sand-pack with brine water, then calculating the porosity of the sand-pack model; (2) measuring the permeability of the sand-pack model with brine water (1% NaCl) at different injection flow rates (0.1, 0.5, 1.0 and 1.5 mL min^−1^) and getting the average permeability; (3) 2 PV (Pore Volume) dispersed swollen fluorescent polymer microspheres solution were injected into the sand-pack model at a rate of 0.5 mL min^−1^, and then injected the brine water at the same injection rate. Recorded the injection pressure and collect the production liquid; (4) measuring the fluorescence intensities at different flooding stages and calculating the concentration of fluorescent polymer microsphere according the relationship between fluorescence intensity and fluorescent polymer microsphere; (5) cleaning the sand-pack model and the pipe lines.

## 3. Results and discussion

### 3.1 Structure characterization of the fluorescent polymer microspheres P(AM-BA-RhB)

**Fig. 4:**
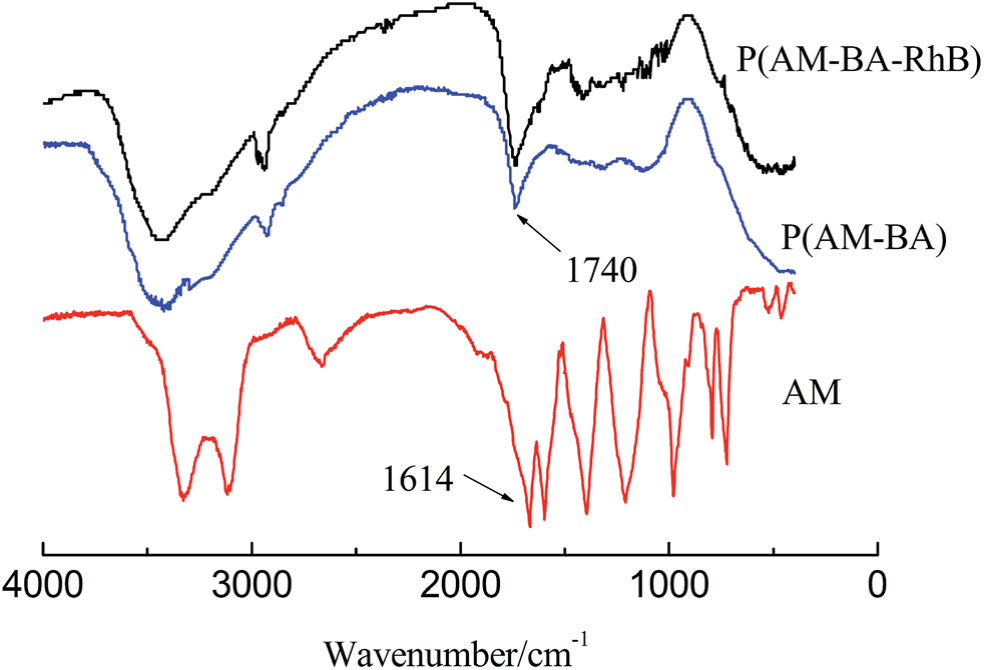
FT-IR spectra of acrylamide and different polymer microspheres.

Fig. 4 shows FT-IR spectra of fluorescent polymer microspheres P(AM-BA-RhB), conventional polymer microspheres P(AM-BA) and acrylamide (AM). The double peak, within the range of 3100 and 3500 cm^−1^ in the spectrum of AM, is attributed to the amide unit and is not available in the spectra of P(AM-BA) and P(AM-BA-RhB). The N–H stretching vibration peak appeared at 3400 cm^−1^, indicating the presence of acrylamide units within the polymer microspheres composed of P(AM-BA) and P(AM-BA-RhB). Furthermore, the C=O bending vibration absorption peak shifted from 1600 cm^−1^ for AM to 1740 cm^−1^ for P(AM-BA), suggesting that the amide is partially hydrolyzed to the carboxyl.^34^ The peak of 670 cm^−1^ is attributed to the bending vibration of aromatic hydrogen. As the trace amount of Allyl Rhodamine B is on the side chains, other characteristic peaks of Allyl Rhodamine B are weak, such as the benzene ring skeleton, methyl and methylene.

**Fig. 5:**
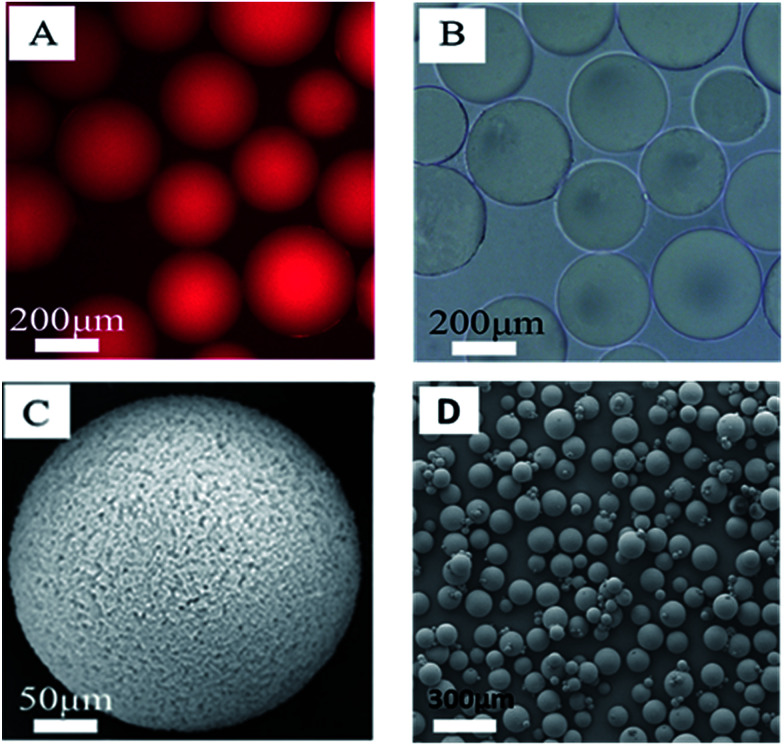
Morphology of the fluorescent polymer microspheres P(AM-BA-RhB): (A) the micrograph of the swollen microspheres under the ultraviolet irradiation with an inverse fluorescence microscope; (B) the micrograph of the swollen microspheres under nature light with an inverse fluorescence microscope; (C) SEM image for microspheres after swelling; (D) SEM image for microspheres before swelling.

**Fig. 6:**
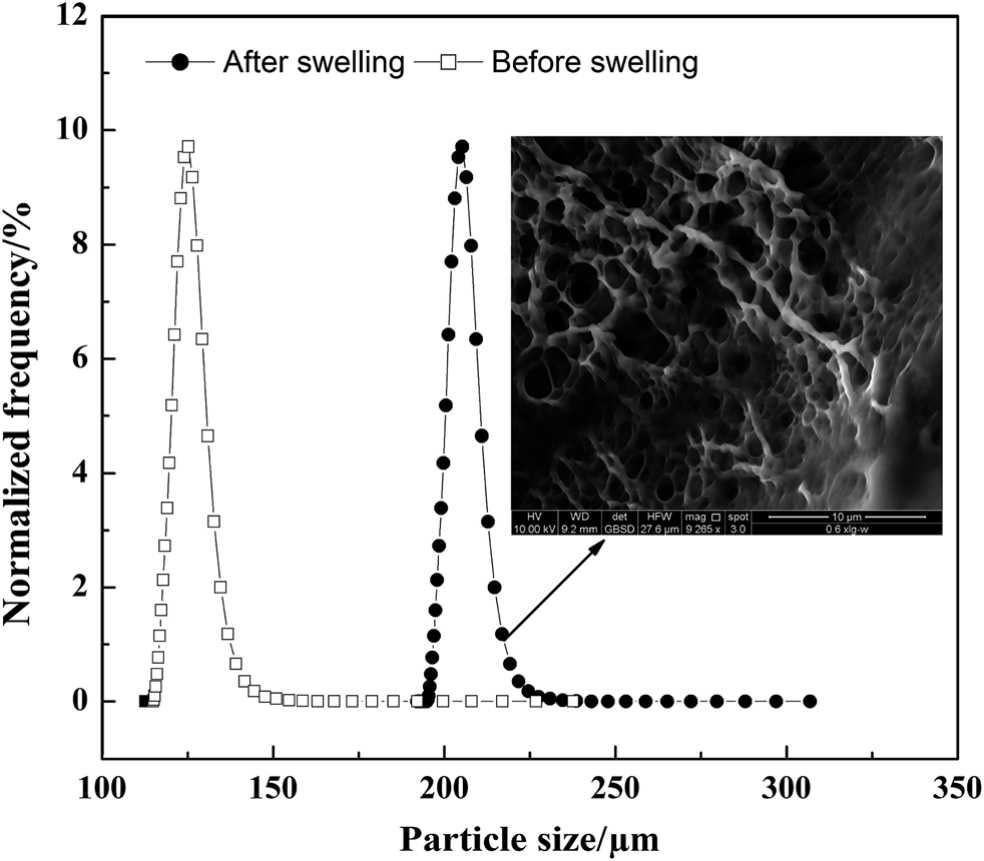
Particle size distribution and three-dimensional cross-linked networks of fluorescent polymer microspheres P(AM-BA-RhB) before and after swelling (1% NaCl, 25 °C).

The morphology of the polymer gel was researched by a fluorescence microscope (Fig. 5A and B), a scanning electron microscope (Fig. 5C and D) and an environment scanning electron microscope (Fig. 6). Fig. 6 gives the particle size analysis and three-dimensional cross-linked networks of fluorescent polymer microspheres P(AM-BA-RhB) before and after swelling at 25 °C. As showed in Fig. 5, the fluorescent polymer microspheres P(AM-BA-RhB) are spherical particles and can swell many times due to absorbing a lot of water (see Fig. 5D and C). As the fluorescent polymer microspheres P(AM-BA-RhB) contact with brine water, the water molecules can go into the internal network under the action of inside and outside osmotic pressure, stretching the molecular chain of the network structure, enlarging the volume of polymer microspheres. The three-dimensional cross-linked networks can be obviously observed in Fig. 5C and 6. It can be seen that the average particle sizes of fluorescent polymer microspheres P(AM-BA-RhB) before and after swelling are 125.7 μm and 215.6 μm, respectively (see Fig. 6). Moreover, from Fig. 5A and B we can see that the swollen polymer microspheres P(AM-BA-RhB) have fluorescent property. The polymer microspheres emit red fluorescence under UV light and the polymer microspheres show some colourless and transparent balls under the ordinary light.

### 3.2 Fluorescent property of fluorescent polymer microspheres P(AM-BA-RhB)

**Fig. 7:**
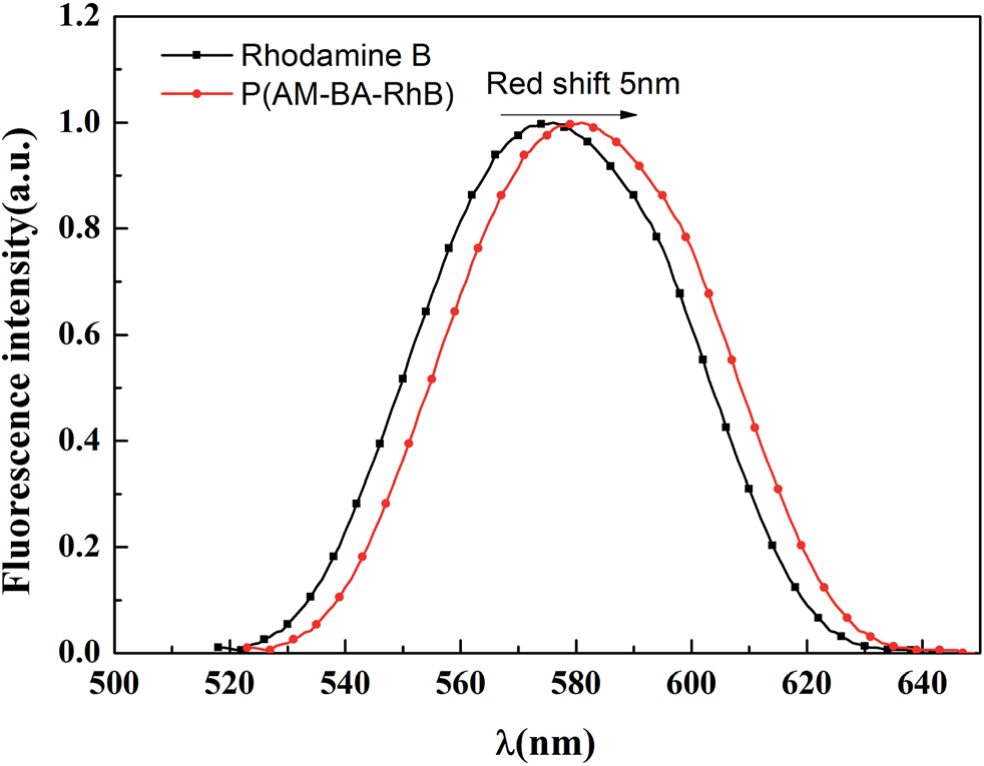
Emission spectra of Rhodamine B and P(AM-BA-RhB) in brine water (*λ*_ex_ = 505 nm).

Fig. 7 shows the fluorescent emission spectra of Rhodamine B and fluorescent polymer microspheres P(AM-BA-RhB) in brine water. It can be observed that the max emission peak of Rhodamine B and P(AM-BA-RhB) were 575 nm and 580 nm, respectively. We find that the emission spectra of fluorescent monomer Rhodamine B moved towards to the long-wavelength band when the Rhodamine B was grafted to the structure of polymer microspheres P(AM-BA-RhB). This means red shift has taken place. The reason why red shift occurred was that when fluorescent monomer Rhodamine B was grafted to polymer microspheres, the conjugation effect of fluorescent monomer Rhodamine B increased, the energy of electron transition decreased and the fluorescence emission wavelength became longer. Martínez *et al.* also found that the fluorescence spectrum will be changed when the fluorescent dye was embedded in the polymer gel by grafting or coating.^35^

**Fig. 8:**
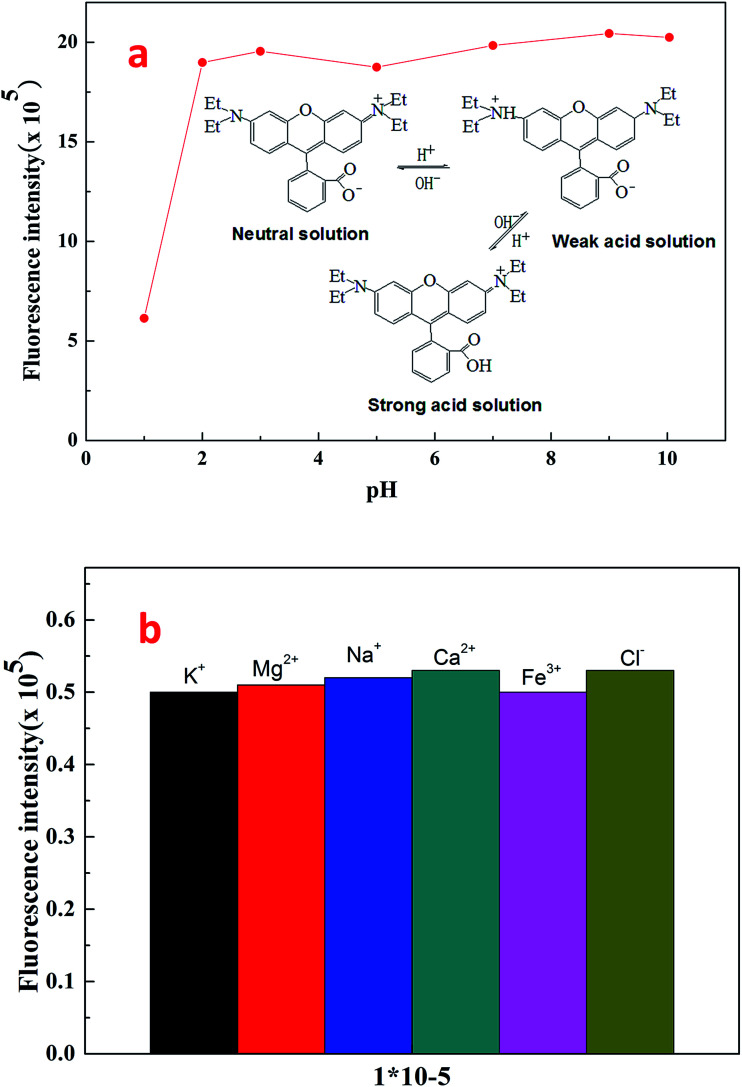
Effect of pH and ionic species on the fluorescence intensity of fluorescent polymer microspheres P(AM-BA-RhB) in brine water.

Fig. 8a shows the effect of pH on the fluorescence intensity of fluorescent polymer microspheres P(AM-BA-RhB) in brine water at the wavelength *λ*_ex_ = 505 nm. When pH < 3, the fluorescence intensity of fluorescent polymer microspheres P(AM-BA-RhB) solution varies with the change of pH. However, the fluorescence intensity remains unchanged when the pH is in the range of 3–10. When pH equals to 1.0, the dispersed solution was strong acid solution and the carboxyl of RhB can't ionize. Because the carboxyl group, which was a strong electron-withdrawing group, may quench the fluorescence of RhB. This caused weak fluorescent property of RhB. The carboxyl of RhB began to ionize and the degree of ionization increased gradually with the increase of pH value. Thus, the electronic absorption ability became weak and the fluorescence enhanced gradually. When the pH value is close to 3.0, the carboxyl of RhB has been completely ionization, the quenching effect disappeared and fluorescence intensity reached the maximum value (see Fig. 8a). When the pH was greater than 3.0, the fluorescence intensity changed little with the increase of pH value. RhB fluorescence intensity presented undulating fluctuation due to the high sensitivity of fluorescence spectrometer. It can conclude that the fluorescent polymer microspheres P(AM-BA-RhB) can be used in the brine water environment with pH greater than 3.0.

Following the general procedure, the effect of ionic species on the fluorescence intensity of fluorescent polymer microspheres P(AM-BA-RhB) was studied (see Fig. 8b). The response of the fluorescent polymer microspheres P(AM-BA-RhB) to solutions containing ionic species such as Na^+^, K^+^, Mg^2+^, Ca^2+^, and Fe^3+^ was investigated. The results indicated that the ionic species had little influence on the fluorescence intensity. When the fluorescent monomer RhB was grafted to the network structure of polymer microspheres, the fluorescence properties were reduced by the influence of the reservoir condition. So the fluorescent polymer microspheres P(AM-BA-RhB) were suitable for the brine water containing these ionic species.

### 3.3 Swelling property of fluorescent polymer microspheres P(AM-BA-RhB)

**Fig. 9:**
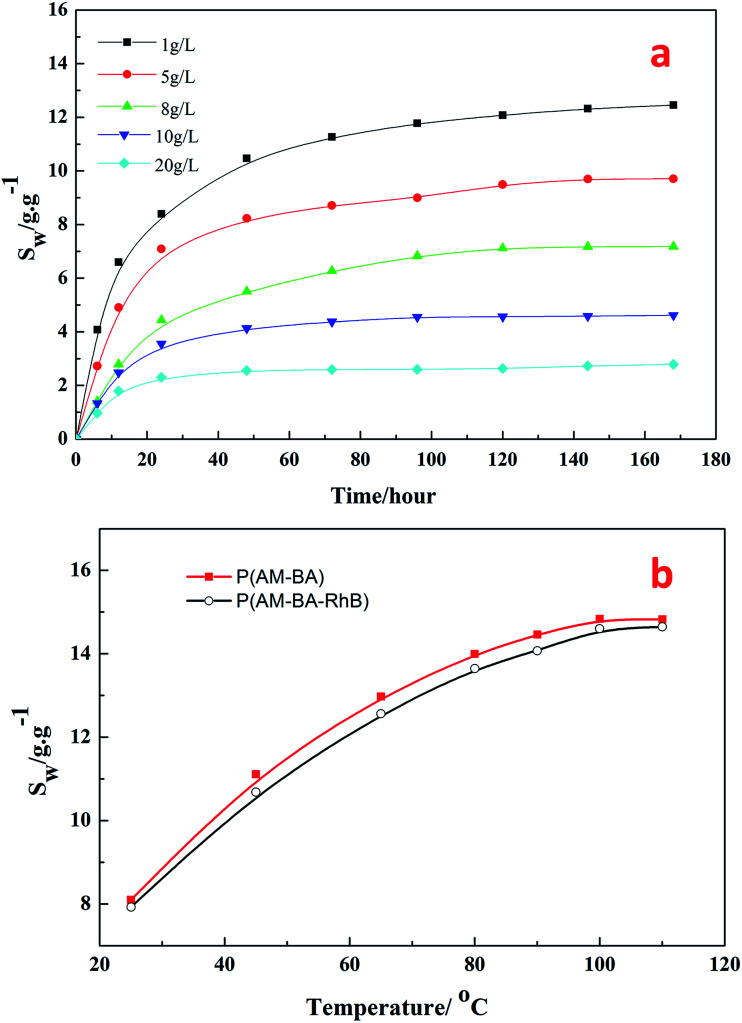
Effects of salinity and temperature on swelling ratio of polymer microspheres.

Fig. 9a shows the swelling ratio of fluorescent polymer microspheres P(AM-BA-RhB) in the brine water with different concentrations of NaCl (1, 5, 8, 10, 20 g L^−1^) at 90 °C. Within 40 hours of hydration, the swelling ratio increased rapidly at an early stage and gradually leveled off. The swelling ratio reached to the maximum after 175 hours. This indicates that brine water can penetrate into the matrix of the fluorescent polymer microspheres. In fact, the fluorescent polymer microspheres are cross-linked particles with a three-dimensional network structure and many free hydrophilic groups (–CONH_2_) inside. Polar brine water molecules can be bond to these groups easily through hydrogen bonding, leading to a significant increase in the hydrated particle size of fluorescent polymer microspheres. While the concentrations of NaCl increased from 1 to 20 g L^−1^, the swelling ratio decreased from 12 to 2 at the equilibrium stage. These results illustrate that the fluorescent polymer microspheres P(AM-BA-RhB) maintain good salt tolerance at high temperature, 90 °C in this case.

Fig. 9b shows the effect of temperature on the swelling ratio of two different polymer microspheres P(AM-BA-RhB) and P(AM-BA) in the brine water with various temperature form 25 to 110 °C. When the temperature increased from 25 to 110 °C, the swelling ratio increased from 8 to 14 times at the equilibrium stage. The two polymer microspheres displayed excellent thermal stability because of the three-dimensional network structures in the polymer microspheres.

Furthermore, there is no obvious difference between the swelling ratios of the two kinds of polymer microspheres (see Fig. 9b). This result suggests that swelling property has not been dramatically affected by the fluorescent functional groups embedded in the polymer microspheres by grafting or coating. Essentially, this result could be attributed to the low content of the fluorescent functional groups.

### 3.4 Method of measuring the concentration of fluorescent polymer microspheres

After studying the swelling property and fluorescence property of fluorescent polymer microspheres, the detection method of measuring the concentration of fluorescent polymer microspheres was discussed in this part. This could provide methods for the research of plugging behavior study of fluorescent polymer microspheres.

#### 3.4.1 Standard curve of measuring the concentration of fluorescent polymer microspheres and detection limit

**Fig. 10:**
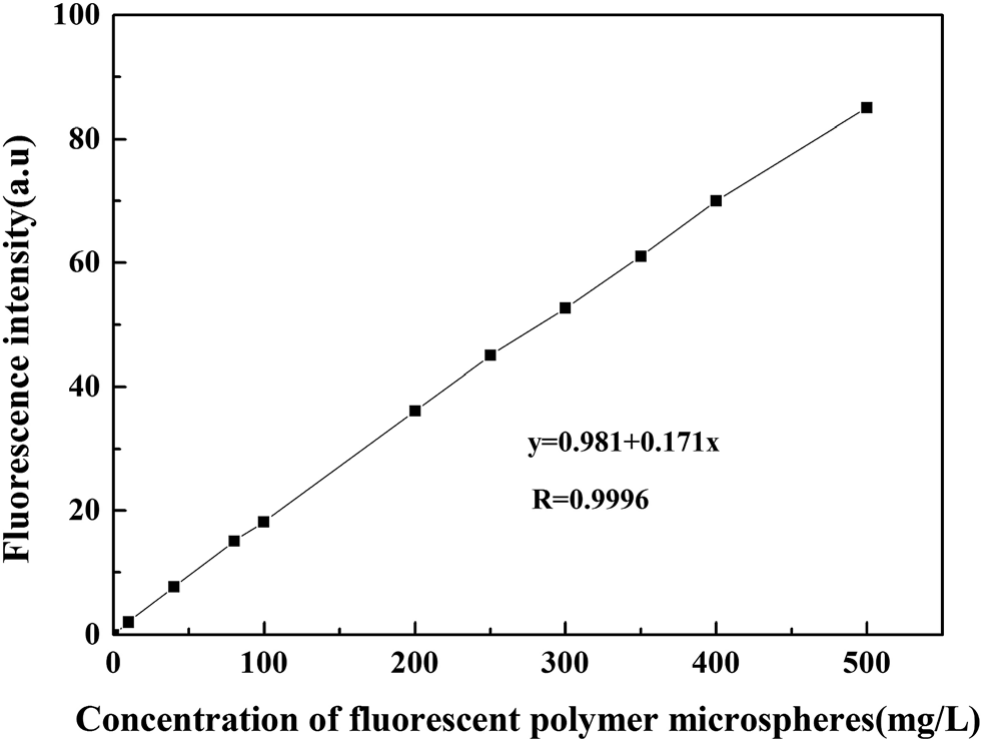
The linear relationship between fluorescence intensity and concentration of fluorescent polymer microspheres P(AM-BA-RhB).

Different concentrations of fluorescent polymer microspheres P(AM-BA-RhB) were moved to quartz sampling cells, the fluorescence emission intensity curve was measured by the scanning slit width of 5 nm. Experimental result of fluorescent polymer microspheres P(AM-BA-RhB) was shown in Fig. 10, the concentration of microspheres had a linear relationship with the fluorescence intensity at the concentration range of 10–500 mg L^−1^. The linear equation was *y* = 0.981 + 0.171*x*, and the correlation coefficient *r* was 0.9997. Carrying out 12 blank determinations and getting the ratio of three times of fluorescence value standard deviation and the slope of the standard curve. The ratio was the detection limit. The detection limit of fluorescent polymer microspheres P(AM-BA-RhB) was 0.5 mg L^−1^. According to this method, the concentration of fluorescent polymer microspheres P(AM-BA-RhB) in the production liquid can be detected quantitatively through the fluorescence intensity value.

#### 3.4.2 Sample determination and accuracy

In order to verify the accuracy of the standard curve, some known concentration of fluorescent polymer microspheres P(AM-BA-RhB) was prepared and then the content of the microspheres was calculated by the standard curve. Compared the actual concentration value and test concentration value of fluorescent microspheres and obtained the relative error. The results were presented in Table 1. As seen from the Table 1, this method is especially accurate in measuring concentration of polymer microspheres with the relative error less than 2% to actual values. This relative error can satisfy the requirements of the measurement.

**Table 1:** The contrast of the actual and test value of the concentration of fluorescent polymer microspheres P(AM-BA-RhB)

Number	Actual value	Test value	Relative error/%
1	15.5	15.3	−1.23
2	30.0	30.4	1.33
3	152.5	153.7	0.79
4	225.0	222.9	−0.93
5	330.5	326.7	−1.15
6	450.0	451.5	0.33

### 3.5 Plugging behavior of fluorescent polymer microspheres

#### 3.5.1 Micro migration and migration mechanism

**Fig. 11:**
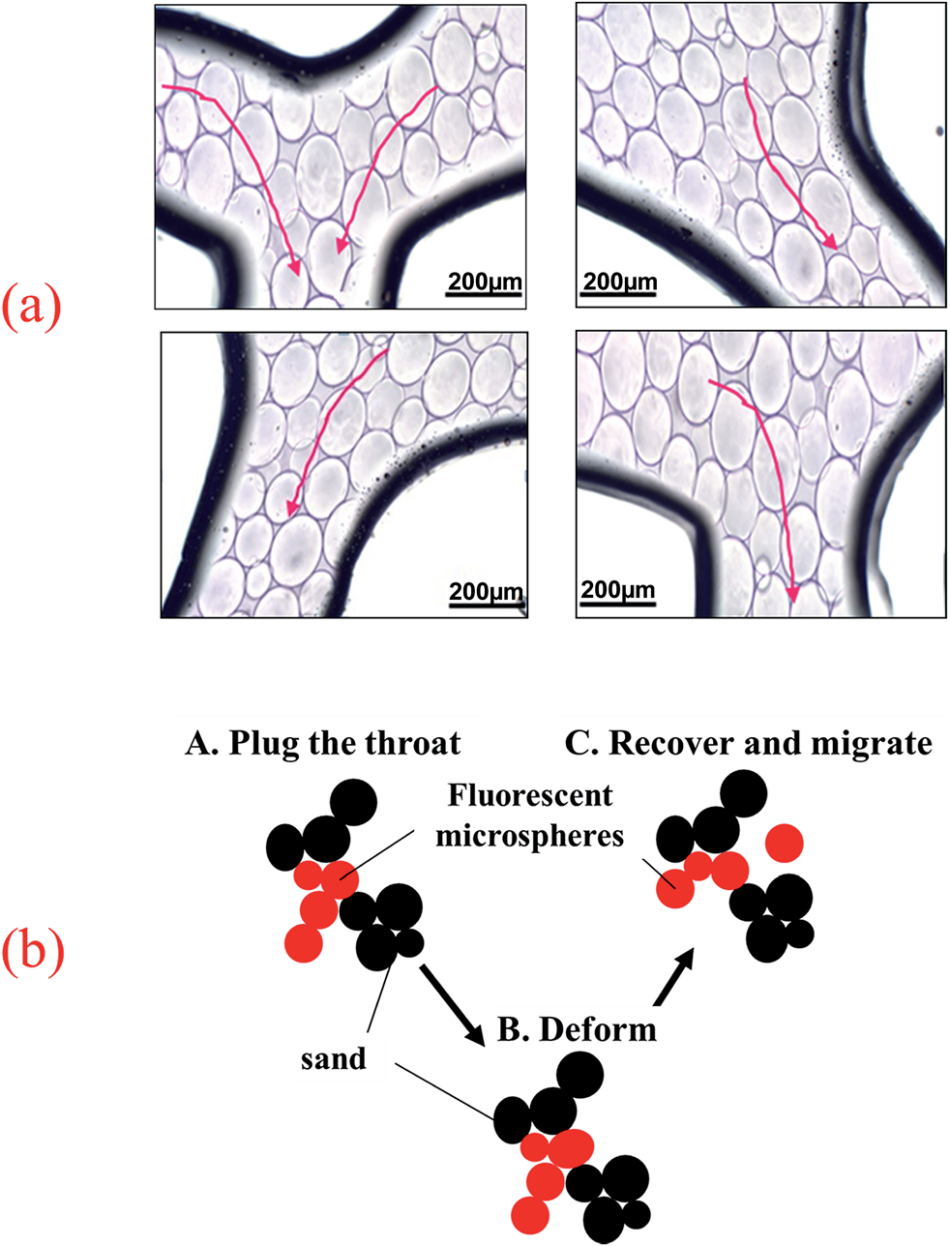
(a) The migration behavior of fluorescent polymer microspheres in the porous medium of visual micromodel and (b) migration schematic diagram.

The visual micromodel was used to simulate channel distribution under reservoir conditions. Fig. 11a illustrates the migration behavior of fluorescent polymer microspheres in the porous medium of visual micromodel. As is shown in Fig. 11a, some fluorescent polymer microspheres were accumulated and squeezed in the pore-throat of the model, playing a role of plugging, so that other fluorescent polymer microspheres cannot move forward, which results in an immobile formation profile being formed under the current pressure. As long as this immobile formation profile is strong enough, following fluorescent polymer microspheres will be blocked by it and then more immobile formation profile will be formed. Plugging effect of the polymer gel to pore-throat is a joint action of those immobile formation profiles. Definitely, the immobile formation profiles are not absolutely immovable. They are dynamically changing. The movement of a small number of fluorescent polymer microspheres will destroy the original layer sand and form new immobile formation profile, *i.e.*, fluorescent polymer microspheres can deform, breakthrough and transport to the next pore-throat so long as sufficient supply of pressure.

Fig. 11b illustrated the migration schematic diagram of fluorescent polymer microspheres. When fluorescent polymer microspheres were being injected, the particles preferentially entered the high permeability zone. Fluorescent polymer microspheres can pass directly or by deformation through the porous media and enter the in-depth formation. When the size of the fluorescent polymer microsphere is much larger than the pore throat size, the fluorescent polymer microspheres will be trapped at the entrance of the pore throat and directly plug the high permeability channel. When the size of fluorescent polymer microsphere is a little larger than the pore throat size but not very much, the polymer gel can be deformed and pass through the pore throat. Moreover, the deformable polymer microspheres can revert to their original shape after entering a larger pore. Additionally, when the size of fluorescent polymer microsphere is smaller than the pore throat size, two or more fluorescent polymer microspheres will be stranded in the pore space and bridge onto the pore throat. Once the bridge is formed and consolidated, the newly arriving fluorescent polymer microspheres accumulate upstream from bridged pores (see Fig. 11b), thus decreasing the following fluid flow rate and yielding fluid diversion effects. When the size of fluorescent polymer microsphere is too smaller than the pore throat size, fluorescent polymer microspheres will easily pass through the pore throat and enter the in-depth formation. In conclusion, the fluorescent polymer microspheres can be deformed and pass through pores easily, so they can enter the in-depth formation and plug the high permeability channels.

#### 3.5.2 Plugging property and plugging mechanism

**Fig. 12:**
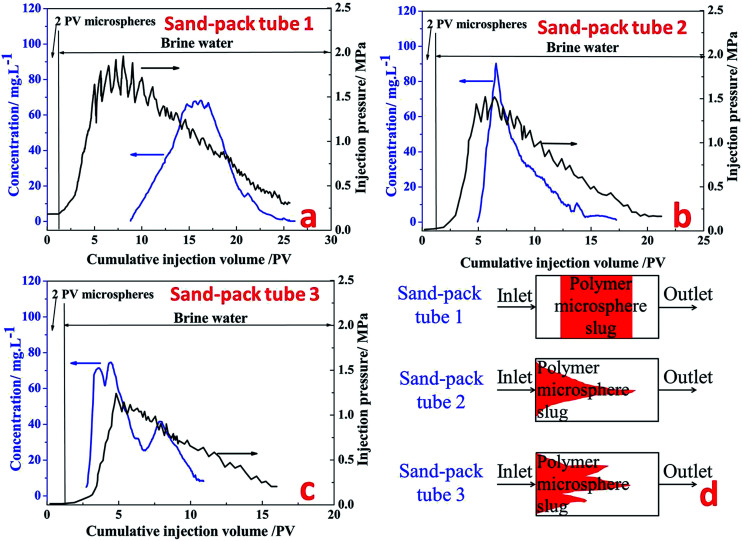
Production concentration, injection pressure curves ((a) tube 1: 12 μm^2^; (b) tube 2: 27 μm^2^; (c) tube 3: 36 μm^2^) and (d) plugging behavior schematic diagram of fluorescent polymer microspheres in the sand-pack tube model.

Three kinds of sand-pack tubes with different permeability were used to study the plugging property of fluorescent microspheres in the porous media. In this part, the concentration of the polymer microspheres was monitored according to the fluorescence intensity of production fluid. According the relationship among permeability, porosity and pore size,^15^ for the sand-pack with permeability of 12, 27, 36 μm^2^ and porosity of 30.8%, 31.5%, 31.9%, the average pore size were 88.2, 130.9, 150.2 μm. The results of the plugging experiments are shown in Fig. 12.

##### Sand-pack tube 1

Fig. 12a shows the production concentration curve and injection pressure curve of fluorescent polymer microspheres in sand-pack tube 1 with permeability of 12 μm^2^. It can be seen from Fig. 12a that the subsequent brine water injection pressure is constantly rising and fluctuating after 2 PV injections of fluorescent polymer microspheres, and finally the injection pressure reaches a peak of 1.9 MPa at 7.3 PV. Then the injection pressure drops slowly in the fluctuation, and at the same time the occurrence of fluorescent microspheres was monitored at the outlet of the tube. With the continuous injection of brine water, the injection pressure begins to decrease slowly, and the concentration of fluorescent polymer microspheres at the outlet increases first and then decreases. Through the observation of the produced concentration curve of the fluorescent polymer microspheres, it can be found that when the injection volume gets to 8–14 PV, the microspheres concentration rises from 0 to 70 mg L^−1^; from 14 PV to 17 PV, the microspheres concentration consists a platform with concentration of 70 mg L^−1^; after 18 PV injection, the fluorescent polymer microspheres concentration begins to decline.

The injection pressure keeps rising in a general trend and drops occasionally within a large range at the brine water injection stage, which is defined as “Wave-type Variation”.^18^ In fact, when the swollen fluorescent polymer microspheres were injected into the tube, they were adsorbed and accumulated on sand surfaces at the beginning. The pressure increased due to blocking pores near the inlet. Moreover, with higher injection volume of fluorescent polymer microspheres, fluorescent polymer microspheres accumulated faster, leading to more rapid pressure increase. Since the swollen fluorescent polymer microspheres have a certain degree of deformation, they could deform under pressure and move to the next pore. This is indicated by the fluctuation when the pressure increased during swollen fluorescent polymer microspheres injection and the pressure continued to rise with large fluctuations.^36^

##### Sand-pack tube 2

Fig. 12b shows the production concentration curve and injection pressure curve of fluorescent polymer microspheres in sand-pack tube 2 with permeability of 27 μm^2^. It can be seen from Fig. 12b that after 2 PV injections of fluorescent polymer microspheres, the subsequent water injection pressure is constantly rising and fluctuating as well, whereas the peak value of injection pressure is 1.5 MPa. Then the injection pressure drops slowly again in the fluctuation, and before the injection pressure getting the peak value, the occurrence of fluorescent polymer microspheres was monitored at the outlet of the tube. Through observation of the produced concentration curve of the microspheres, we can find that the concentration curve of fluorescent polymer microspheres shows a sharp peak. It is different from the production curve in Fig. 12a.

##### Sand-pack tube 3

Fig. 12c shows the production concentration curve and injection pressure curve of fluorescent polymer microspheres in sand-pack tube 3 with permeability of 36 μm^2^. It can be seen from Fig. 12c that the subsequent water injection pressure is constantly rising with fluctuation as well after 2 PV injections of fluorescent polymer microspheres, whereas the peak value of injection pressure is 1.25 MPa. Then the injection pressure drops slowly again in the fluctuation, and before the injection pressure getting the peak value, the occurrence of fluorescent polymer microspheres was also monitored at the outlet of the tube. Through observation of the produced concentration curve of the microspheres, it is found that the concentration curve of fluorescent polymer microspheres shows three sharp peaks. It is different from the production curves in Fig. 12a and b.

It is noteworthy that the fluorescent polymer microspheres production concentration reaches a platform from 14 PV to 17 PV in the sand-pack tube 1, which indicates that the fluorescent polymer microspheres are produced at a certain high-concentration for a period of time. And it is assumed that the microspheres mainly move parallel to the piston in the sand-pack tube 1 (see Fig. 12d). This is named as “piston plugging”. In the sand-pack tube 2, the concentration curve of fluorescent polymer microspheres shows one peak at 6 PV, indicating that the fluorescent polymer microspheres move along a large-pore channel, and this is called “protruding plugging” (see Fig. 12d). However, in the sand-pack tube 3, there are three peaks in the production concentration of fluorescent polymer microspheres, indicating that the fluorescent polymer microspheres are transported through three large channels, which is called “fingering plugging” (see Fig. 12d). Due to the difference of fracture distribution in the reservoirs, fluorescent polymer microspheres have different plugging behavior. The slug design of polymer microspheres should fully consider the plugging behavior in the reservoirs.

## 4. Conclusions

In this study, a kind of fluorescent polymer microspheres P(AM-BA-RhB) acted as a novel conformance control agent was proposed. Laboratory experiments have been conducted to understand the structure characterization, fluorescent property, swelling property and plugging behavior of fluorescent polymer microspheres. The major conclusions that can be drawn from this study are as follows:

(1) The fluorescent polymer microspheres P(AM-BA-RhB), which can fluoresce under ultraviolet irradiation, was synthesized *via* the inverse suspension polymerization method. The fluorescence functional monomer of Rhodamine B was uniformly distributed inside the polymer microspheres. When the Rhodamine B was grafted on the structure of polymer microspheres P(AM-BA-RhB), the red shift occurred. The fluorescent polymer microspheres retained their fluorescent properties after swelling in the metal ions containing solution at the pH 3.0–10.0.

(2) The fluorescent polymer microspheres P(AM-BA-RhB) were spherical particles with an average particle size of 125.7 μm. This kind of microspheres was composed of three-dimensional cross-linked networks and had good swelling property in high salinity and high temperature conditions. With the increase of salinity, the swelling ratio decreased. And the swelling ratio increased with the increase of the formation temperature. The swelling property was not dramatically affected by the fluorescent functional groups embedded in the polymer microspheres by grafting or coating.

(3) The linear equation between fluorescence intensity and concentration of fluorescent polymer microspheres P(AM-BA-RhB) was constructed. According to this linear equation, the concentration of fluorescent polymer microspheres in the production liquid can be detected quantitatively through the fluorescence intensity value.

(4) Visual micromodel test and sand-pack tubes experiment of fluorescent polymer microspheres showed that the fluorescent polymer microspheres can pass directly or by deformation through porous media and enter the in-depth formation. The injection pressure showed the phenomenon of “Wave-type Variation”. Three plugging behaviors such as piston plugging, protruding plugging and fingering plugging were put forward. These results were useful for the slug design of polymer microspheres in the field application.

## Conflicts of interest

The authors declare no competing financial interest.

## Supplementary Material
